# A Physiologic Approach to Hemodynamic Monitoring and Optimizing Oxygen Delivery in Shock Resuscitation

**DOI:** 10.3390/jcm9072052

**Published:** 2020-06-30

**Authors:** Amy Russell, Emanuel P. Rivers, Paresh C. Giri, Anja K. Jaehne, H. Bryant Nguyen

**Affiliations:** 1 Division of Pulmonary, Critical Care, Hyperbaric, Allergy, and Sleep Medicine, Loma Linda University, Loma Linda, CA 92354, USA; PGiri@llu.edu; 2 Department of Emergency Medicine, Loma Linda University, Loma Linda, CA 92354, USA; ARussell@llu.edu; 3 Department of Emergency Medicine, Henry Ford Hospital, Detroit, MI 48202, USA; ERivers1@hfhs.org (E.P.R.); AJaehne2@hfhs.org (A.K.J.); 4 Surgical Critical Care, Henry Ford Hospital, Detroit, MI 48202, USA

**Keywords:** shock, resuscitation, hemodynamic monitoring, fluid responsiveness, stroke volume, venous oxygen saturation, lactate, oxygen delivery

## Abstract

The approach to shock resuscitation focuses on all components of oxygen delivery, including preload, afterload, contractility, hemoglobin, and oxygen saturation. Resuscitation focused solely on preload and fluid responsiveness minimizes other key elements, resulting in suboptimal patient care. This review will provide a physiologic and practical approach for the optimization of oxygen delivery utilizing available hemodynamic monitoring technologies. Venous oxygen saturation (SvO_2_) and lactate will be discussed as indicators of shock states and endpoints of resuscitation within the framework of resolving oxygen deficit and oxygen debt.

## 1. Introduction

Circulatory shock is defined as inadequate oxygen delivery to meet metabolic and oxygen demands [1]. Mechanisms underlying this physiologic emergency are related to decreased cardiac output (CO) due to either decreased circulating volume (hypovolemic shock), obstruction of circulatory flow (obstructive shock), or impaired cardiac function (cardiogenic shock). A fourth recognized mechanism is based on the altered distribution of blood flow leading to perfusion failure (distributive shock). The occurring oxygen debt and tissue hypoperfusion, regardless of the underlying etiology of the shock, lead to cellular ischemia and injury and potentially to multi-organ dysfunction syndrome (MODS) and eventual death.

**Figure 1:**
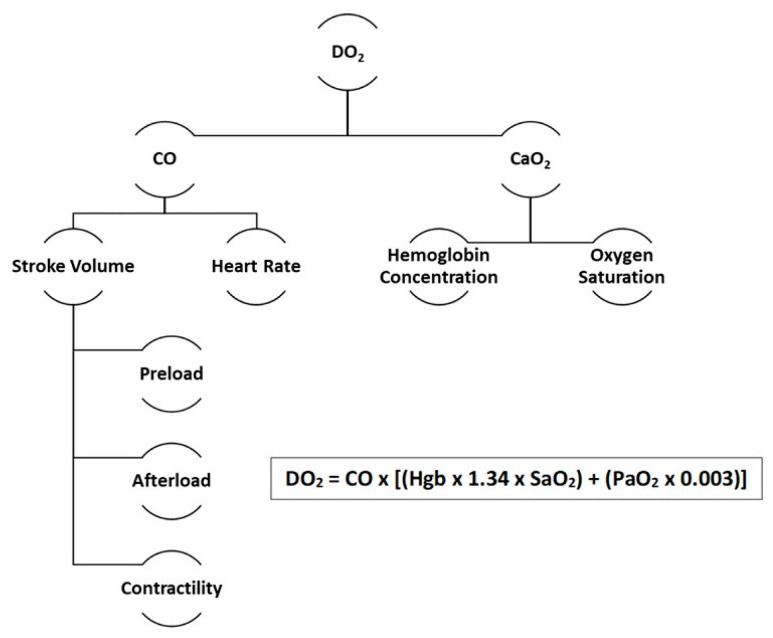
Determinants of oxygen delivery (DO_2_). CO–Cardiac output; CaO_2_–Oxygen content; Hgb–Hemoglobin concentration; SaO_2_–Oxygen saturation; PaO_2_–Partial pressure of oxygen.

By its definition, the goal in the treatment of shock is, therefore, to increase oxygen delivery (DO_2_) to meet oxygen demand in order to resolve the global tissue hypoperfusion. Oxygen delivery is determined by CO and blood oxygen content (CaO_2_) (Figure 1). Therefore, resuscitation of shock at the bedside is dependent on optimizing the components of CO and CaO_2_: Preload, afterload, contractility, hemoglobin, and oxygen saturation. 

Unfortunately, recent literature discussing shock resuscitation focuses only on preload, specifically fluid responsiveness [2,3,4]. The passive leg raise (PLR) maneuver has been popularized as a non-invasive method of simulating a fluid bolus due to fears of administrating an actual bolus [5]. Additionally, there has been much debate regarding what amounts to a fluid bolus and what type of fluid should be used.

In this review, we will provide the reader with a practical review on shock resuscitation beyond the fluid bolus and assessment of fluid responsiveness, discussing the physiologic basis for various bedside hemodynamic monitoring tools available to optimize oxygen delivery. While fundamental, we believe that these concepts are important for clinicians to apply when faced with patients having complex hemodynamic presentations. We will first discuss measurements of preload as this is the initial target of resuscitation. We then review the techniques of determining fluid responsiveness based on the classical Frank–Starling and Guyton curves. Optimizing preload thus requires measuring stroke volume (SV) and CO with a number of available technologies. Once preload is addressed, afterload and contractility are targeted. Finally, the resuscitation endpoints venous oxygen saturation (SvO_2_) and lactate are applied to determine if shock persists, requiring further increased oxygen delivery with inotrope, blood transfusion, and/or oxygen supplementation (e.g., mechanical ventilation support).

## 2. Measuring Preload

### 2.1. Left Ventricular End-Diastolic Volume and Pressure

Preload is the first target for the resolution of shock. Left ventricular end-diastolic volume (LVEDV) is the reference standard for the assessment of preload. LVEDV can be measured or estimated using either transthoracic echocardiography (TTE) or transesophageal echocardiography (TEE). The apical four-chamber view visualizes the left ventricle. The LVEDV measurement is based on images obtained in end-diastole corresponding with the R-wave on an electrocardiogram. LVEDV is calculated based on tracing the endocardium at end-diastole and applying a modified Simpson’s rule [6]. This assessment is user-dependent with respect to skill and appropriate image acquisition. Additionally, LVEDV is measured at a singular time point, not addressing the need for serial assessments required to evaluate response to interventions. Alternatively, pressure instead of volume is often measured, which has its own set of limitations. Left ventricular end-diastolic pressure (LVEDP) measured by the left heart catheterization can be a false representation of LVEDV in common pathological states such as chronic left heart failure with low compliance. Compliance is equal to the change in volume divided by the change in pressure (C = ΔV/ΔP). With a decrease in ventricular compliance (stiffening of the ventricle) seen in left ventricular hypertrophy, the left ventricular pressure can be elevated in the presence of hypovolemia.

### 2.2. Pulmonary Capillary Wedge Pressure and Left Atrial Pressure

Using a pulmonary artery catheter (PAC) we can measure left atrial pressure (LAP) via pulmonary capillary wedge pressure (PCWP) as a surrogate. A balloon-tipped catheter is introduced and advanced to the pulmonary arteries, where it can be wedged. The column of blood between the catheter tip and left atrium reflects a pressure that can be measured [7]. The PAC may also be used to measure pulmonary artery diastolic pressure (PADP) and right ventricle pressure. Because the pulmonary artery catheter is invasive, it should be limited to critically ill patients with complex physiology such as refractory shock with advanced right heart failure. Risks include infection, perforation, thrombus/embolism, arrhythmia, and balloon rupture [8]. Use of PAC in the routine setting has not been proven to improve outcomes [9,10]. 

### 2.3. Central Venous Pressure and Right Atrial Pressure

**Figure 2:**
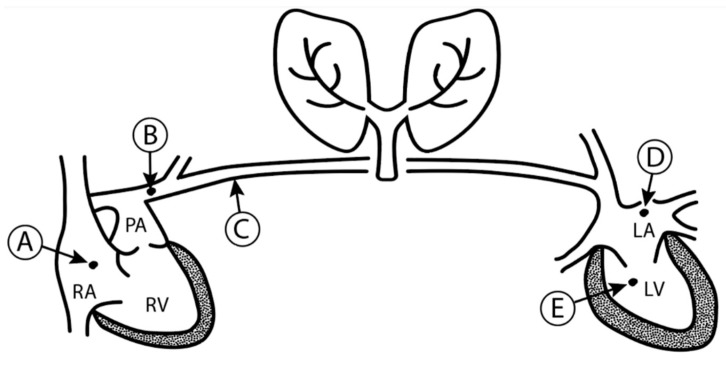
Pressure Relationships. A patient can have falsely elevated central venous pressure (A) in the setting of any of the following: Left ventricular hypertrophy affecting left ventricular compliance (E), mitral valve disease increasing left atrial pressure (D), pulmonary disease increasing alveolar pressure (C), pulmonary artery disease increasing pulmonary artery pressure (B), right ventricular hypertrophy affecting compliance of the right heart or tricuspid disease increasing right atrial pressure (A).

Historically, one of the first methods used to measure preload was via central venous catheterization to measure central venous pressure (CVP) and right atrial pressures (RAP). Measuring CVP or right ventricular preload has been repeatedly proven to be a poor indicator of intravascular volume [11]. Inaccuracies are rooted in the concept that, as with all measurements of pressure as a surrogate to volume, many parameters must be normal for CVP to be accurate and useful. Patients must have normal right ventricular compliance, no pulmonary vascular disease, no valvular heart disease, and no left ventricular failure (Figure 2).

## 3. Determining Fluid Responsiveness

### 3.1. The Frank–Starling Law and Guyton’s Venous Return

The purpose of measuring preload is based on the fundamental concept of Frank–Starling physiology. The Frank–Starling curve describes the relationship between cardiac muscle fibers and the force that is produced with the contraction of these fibers. Sarcomeres (the functional units of myofibrils) generate the most force when stretched to an optimal length. The stretching of myocardial fibers occurs during diastole, and thus the preload (end-diastolic volume) determines the length to which those myocardial fibers are stretched. If either over- or under-stretched, the myocardial fibers cannot generate an optimal force [12]. In states of fluid overload, as commonly seen in patients with congestive heart failure, myocardial fibers are overstretched at end-diastole and, therefore, do not generate an optimal force. This results in reduced SV and CO. In states of intravascular volume depletion, as often seen in sepsis patients, myocardial fibers are under stretched. 

**Figure 3:**
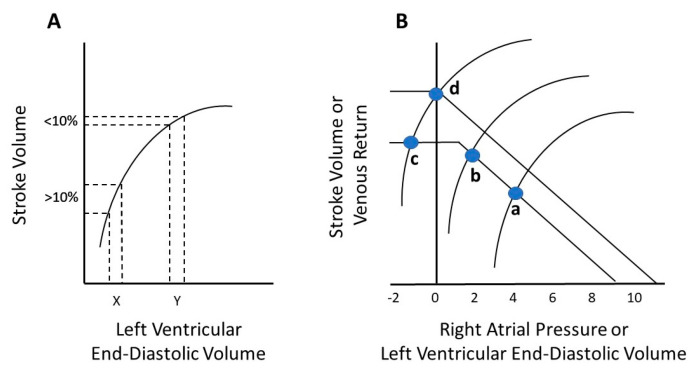
Frank–Starling curve (**A**). An increase in stroke volume of >10% after a fluid bolus is indicative of fluid responsiveness (X). When stroke volume increases <10% in response to fluid administration (Y), further fluid resuscitation is not indicated, and the “peak of the Frank–Starling curve” is reached. Guyton’s venous return curve interposed on Frank–Starling curve (**B**). Point (a) is the intersection of the venous return and stroke volume curves referred to as the hemodynamic “operating point” of the system. A leftward shift of the Frank–Starling curve from (a) to (b) with an inotrope results in a higher stroke volume and a lower right atrial pressure (RAP). A decrease in RAP to sub-atmospheric levels causes collapse of the great veins entering the thorax and prevents stroke volume and venous return from increasing (c), despite the further leftward shift of the Frank–Starling curve. The addition of fluids could move the operating point from (c) to (d) by increasing the stressed volume and mean systemic filling pressure, which is the upstream pressure for venous return to the right heart.

Patients are considered to be “fluid responsive” if SV increases more than 10% after administering a fluid bolus (Figure 3A). The delivery of fluids improves cardiac performance by shifting to a more favorable point on the Frank–Starling curve. In practice, fluids are given until there is no longer a greater than 10% increase in SV [13]. In cases in which there is a baseline decreased cardiac contractility such as heart failure, the Frank–Starling curve is shifted downward, and adding an inotrope allows for an upward shift of the curve. In this situation, preload can be further increased after inotrope administration. To illustrate this physiology, Guyton proposed interpolating the venous return curve to the classic Frank–Starling curve (Figure 3B) [14]. At the bedside, a decrease in CVP and/or PCWP may indicate that the addition of inotrope was effective, and perhaps more fluids are needed. Determining fluid responsiveness using various technologies requires an understanding of the Frank–Starling cardiac function and Guyton venous return principles.

### 3.2. The Classical Fluid Bolus

There are multiple ways to determine fluid responsiveness. As discussed, the traditional method based on the Frank–Starling curve is to administer a fluid bolus and determine if SV increases more than 10%. However, there is much debate on what determines a fluid bolus and how much fluid to give. Glassford et al. completed a study in which critical care specialists in 30 countries participated in a practice-based survey to answer what is the definition and expectations of a fluid bolus [15]. More than 80% of clinicians indicated that a fluid bolus is more than 250 mL of either colloid or crystalloid given over less than 30 min, which resulted in a variety of physiological responses such as increased mean arterial pressure, decreased heart rate, increased urine output, and other responses. While common, “more than 250 mL” of “either colloid or crystalloid” has been a non-specific application of Frank–Starling law with continuing debate. 

Crystalloids, specifically Ringer’s lactate or isotonic saline, are commonly selected for initial fluid resuscitation in the hypotensive patient. A pitfall of this fluid selection is that the volume expanding effects are transient. In comparison, colloids are known to optimize the amount of fluid that remains in the intravascular space and thus can restore volume faster and for a longer amount of time [16]. The CRISTAL trial did not show a difference in 28-day mortality between patients given crystalloids versus colloids in critically ill patients with hypovolemia, however, there were fewer deaths at 90 days in the colloid group [17]. For septic shock, the Surviving Sepsis Campaign guidelines suggest the addition of albumin when patients require significant amounts of crystalloids during resuscitation but recommend against the use of the hydroxyethyl starches as these may increase the risk of acute kidney injury and death [18].

### 3.3. The 5-2-0 Rule 

While an absolute CVP target is not recommended, the late Dr. Max Harry Weil first described the 5-2-0 rule using CVP to evaluate fluid responsiveness [19]. This technique involves first observing CVP for 10 min and then delivering a fluid bolus based on the initial CVP. If CVP < 8 cm H_2_O, then a 200 mL bolus is administered over 10 min. If CVP ≤ 14 cm H_2_O, then a 100mL bolus is administered, and if CVP > 14 cm H_2_O, a 50 mL bolus is given. During minutes 0–9 of infusion, if CVP increases by >5 cm H_2_O, then infusion is stopped. After infusion is complete, if CVP increases by 2–5 cm H_2_O then fluids are stopped. If CVP increases by <2 cm H_2_O, then infusion is continued as the patient is still fluid responsive.

### 3.4. Inferior Vena Cava (IVC) Collapsibility

Targeting CVP and SV have required inserting a central venous or pulmonary artery catheter, which may be impractical, especially when a patient first presents in the emergency department. Thus, clinicians have trended towards utilizing inferior vena cava (IVC) collapsibility as a bedside method for determining fluid responsiveness. Using ultrasound, fluid status can be evaluated by measuring the IVC diameter [20,21].

When the IVC is under-filled, there is a greater compliance of the vessel, resulting in a collapse during spontaneous inspiration. In hypovolemic states, the pressure gradient between the thoracic portion of the IVC and abdominal portion of the IVC is greatest, which forces blood out of the IVC and into the right atrium. Typically, an IVC diameter ≤ 2.1 cm and > 50% collapsible correlates with CVP 0–5 mm Hg. IVC diameter > 2.1 cm and < 50% collapsible correlates to CVP 10–20 mm Hg [22]. Limitations of this technique, as with any ultrasound application, include operator expertise and variability when obtaining IVC images. Additionally, the same physiologic limitations of CVP as a measure of preload apply to the IVC since “normal” heart–lung parameters are often not present in critically ill patients. 

### 3.5. Passive Leg Raise (PLR)

The passive leg raise (PLR) maneuver provides an artificial fluid bolus, and thus reduces the risk of inappropriate administration of fluids in patients who are not fluid responsive. This technique involves raising the patient’s leg from flat to 45° position for 5 min in order to transfer blood from the lower extremities to the thoracic cavity and approximates a 200–300 mL fluid bolus. Bentzer et al. performed a meta-analysis analyzing 2260 patients and found that PLR effectively identified patients whose CO would be augmented by the administration of a fluid bolus (pooled specificity, 92%) and PLR also effectively identified those patients who would not be fluid responsive (pooled sensitivity, 88%) [3]. However, while there is significant data to support the accuracy of the PLR for predicting fluid responsiveness, Chopra et al. sought to examine the precision of predicting fluid responsiveness with PLR determined by the non-invasive cardiac output monitoring technology, NiCOM™ (Cheetah Medical, Inc., Newton Center, MA 02459, USA) [23]. Their study found a 9% standard deviation in the precision of determining the change in SV after the PLR maneuver in both critically ill patients and healthy volunteers. Their results raised the question of having a strict cutoff SV increase by 10% to identify fluid responsiveness.

### 3.6. Stroke Volume Variation (SVV) and Pulse Pressure Variation (PPV)

Taking into account the heart–lung interactions to determine fluid responsiveness may avoid the need to perform a maneuver such as PLR or to administer a potentially harmful fluid bolus. Spontaneously breathing patients who are hypovolemic will have a greater reduction in SV and blood pressure at the end of inspiration, similar to pulsus paradox in patients with cardiac tamponade (or obstructive shock). In the setting of mechanical ventilation, as positive pressure is introduced to the thorax, there is a rise in pleural pressure, which decreases venous return and leads to a reduction in right ventricle preload. Right ventricular afterload (increased pulmonary vascular resistance) also increases, resulting in decreased right ventricular stroke volume. Conversely, the increase in transpulmonary pressure is thought to increase left ventricular preload as blood is essentially squeezed out of the pulmonary vasculature and into the left ventricle. Left ventricular afterload decreases because positive pleural pressure causes a decrease in thoracic blood volume; therefore, resulting in an increased left ventricular stroke volume during inspiration. 

Because of the approximately two-second pulmonary transit time, the inspiratory decrease in right ventricular stroke volume causes a decrease in left ventricular stroke volume a few heart beats later during expiration [24,25]. Recognizing this physiology, stroke volume variation (SVV) or pulse pressure variation (PPV) is the difference between the maximal (inspiratory) and minimal (expiratory) stroke volume or pulse pressure, respectively, over 3 to 5 breaths divided by the average [8]. Studies have shown that SVV or PPV > 10–12% is indicative of fluid responsiveness; i.e., SV increase >15% in response to a fluid bolus administration [26,27].

PPV can be measured via arterial based systems and derived through arterial waveform analysis. SVV is computed by proprietary algorithms via the area under the systolic pressure curve. Limitations of SVV and PPV measurements are that they have only been validated in patients without arrhythmias, on mechanical ventilation without spontaneous respiration and with high tidal volumes > 8 mL/kg [28].

## 4. Measuring Stroke Volume and Cardiac Output

### 4.1. Indicator Dilution Methods

If the purpose of increasing preload is to increase SV, then we must have bedside techniques to measure changes in SV after fluid administration. Thermodilution via the pulmonary artery catheter is considered the “gold standard” in SV and CO measuring techniques. This method is used to derive SV based on how rapidly a cold injectate warms (or becomes diluted) over time. Boluses of ice-cold fluid are injected into the right atrium, and blood temperature is measured by a thermal filament on the catheter in the pulmonary artery and used to calculate SV [7,29]. A similar technique uses a lithium (instead of cold fluid) injectate into the central venous catheter. A lithium sensitive sensor attached to a peripheral arterial line then detects the concentration of lithium ions in the arterial blood; the “wash-out curve” is then used to derive SV. 

### 4.2. Arterial Waveform/Pulse Contour Analysis

Given the invasiveness of inserting a pulmonary artery catheter to measure SV and CO, a minimally invasive method of analyzing the continuous artery pressure waveform has become popular in recent years. These systems use blood flow sensors connected to an arterial line that analyzes arterial pulsatility and converts pressure-base signals into flow measurements from which SV and CO can be derived. Importantly, several technologies have been developed to calculate SVV and PPV based on the information derived from arterial waveform analysis, therefore, making this hemodynamic monitoring technique attractive [30].

However, there is ongoing debate surrounding the accuracy and outcome benefits provided by this technology. CO measurements may be “uncalibrated,” which indicates the values are calibrated to biometric or physiological data from large patient datasets. “Calibrated” values are calibrated to an external reference measurement of CO, such as indicator dilution methods in the same patient. Regardless of the calibration method, it has been shown that CO measurements by pulse contour analysis are inaccurate in situations of hemodynamic stability. Therefore, the timing for recalibration is crucial and should be made on a case by case basis [31]. Peyton et al. performed a pool-weighted meta-analysis of 4 methods of cardiac output measurements, including pulse contour analysis, and found that all of the methods lacked the percentage error limit of 30% in agreement with thermodilution measured CO [32].

### 4.3. Transthoracic (TTE) and Transesophageal Echocardiography (TEE) 

SV can be calculated by multiplying the area of the aortic valve by the velocity–time integral of a Doppler signal across the left ventricular outflow tract; essentially, volume = area × distance. This can be accomplished with either transthoracic or transesophageal echocardiography. Limitations are similar to those mentioned above for measuring LVEDV. Limitations of TEE include esophageal intubation with a flexible probe, which is not well tolerated in patients who are awake. Additionally, the probe must be properly positioned before each measurement [33]. 

### 4.4. Bioimpedance and Bioreactance

The concept of bioimpedance can be understood in terms of Ohm’s law, where the flow of current (I) is equal to the voltage (V) drop between the two ends of a circuit, divided by the impedance or resistance (R) (I = V/R). Impedance is related to changes in volume, and these changes in volume can be used to calculate SV. As blood flows through the thorax with each cardiac cycle, a high-frequency, low-magnitude current is passed across the thorax, which can be measured with electrodes. Stroke volume can then be calculated using mathematical algorithms. Bioreactance was developed to measure changes in the frequency of the electric currents; the advantage of this being the reduction of background noise [34]. Limitations include the requirement of proper patient positioning and electrode placement for accurate measurements. Critically ill patients may have multiple factors contributing to changes in thoracic impedance, and thus these techniques are likely more accurate in relatively stable patients [35,36,37,38].

### 4.5. Transcutaneous/Transesophageal Doppler

The principle of the Doppler effect is based on the flow velocity of red blood cells. As blood flows through a vessel, RBCs emit and reflect ultrasound waves. The difference between the frequencies of these waves is proportional to the velocity of the red blood cells. An ultrasound probe placed at the sternal notch can measure Doppler flow across the aortic or pulmonary valve to measure SV and CO. Some studies suggest this method is reliable regardless of heart rhythm, ventilator, or vasoactive agents, and thus can be considered ideal for non-invasive monitoring. Other studies suggest a lack of correlation with standard TTE [39,40,41]. Advantages include good interrater reliability regardless of the level of training [42,43]. 

Alternatively, a transesophageal Doppler method can be used for continuous monitoring of SV and CO with an esophageal probe placed adjacent to the descending aorta. This technique has been validated by multiple studies, including surgical patients and those receiving positive pressure ventilation [44,45]. Limitations include the need for the Doppler signal to be continuously measured at the same angle with frequent adjustments at the bedside. 

## 5. Optimizing Afterload

We often use mean arterial pressure (MAP) as a surrogate for afterload, keeping in mind that MAP = CO × systemic vascular resistance (SVR). Once we have optimized SV and CO to the plateau region of the Frank–Starling curve, then low MAP must indicate low SVR. Therefore, in cases of septic shock, vasopressor agents may be required to increase SVR. Conversely, in cases of cardiogenic shock or acute heart failure, SVR may be high and vasodilator agents can be used to decrease afterload and increase CO. Based on the Surviving Sepsis Guidelines, the first-line vasopressor used for patients in septic shock should be norepinephrine [46]. Second-line agents include vasopressin and epinephrine, when norepinephrine is not sufficient to achieve MAP goals. Vasopressin should be used at 0.03 U/min and should not be used as the only vasopressor. Because epinephrine stimulates anaerobic metabolism in skeletal muscle, it may increase lactate levels rendering lactate a less ideal indicator of hypoperfusion. Phenylephrine should only be used as a third-line agent in septic shock. Its side effect of bradycardia may be helpful in patients with severe tachycardia or at risk for tachyarrhythmia, such as atrial fibrillation. Dobutamine may be used for patients having low CO while on vasopressors with preserved MAP, but still showing evidence of inadequate perfusion.

Afterload reducing agents (vasodilators) may be used in the setting of heart failure or cardiogenic shock. In patients with acute decompensated heart failure, intravenous nitrates are first-line agents. Nitroprusside decreases preload and afterload and is useful in patients with severe hypertension. The metabolite of nitroprusside is cyanide and thus majorly limits its use. Nitroglycerin reduces preload more than afterload and is ideal in patients with heart failure and low CO. Other afterload reducing agents include intravenous hydralazine, which should be used cautiously as it may cause reflex tachycardia, and is not ideal in patients with underlying coronary artery disease or aortic dissection. Enalaprilat is an angiotensin-converting enzyme inhibitor in intravenous form that is another afterload reducing agent. It should be avoided in patients with acute myocardial infarction, hyperkalemia, and renal artery stenosis [47,48]. 

In the critically ill patient, a target MAP goal of 65–75 mm Hg is appropriate. For septic shock patients undergoing resuscitation, there is no mortality difference in targeting MAP 65–70 mm Hg versus 80–85 mm Hg [49]. Recently, the COMACARE trial compared low normal MAP (65–75 mm Hg) with high normal MAP targets (80–100 mm Hg) in post-cardiac arrest and saw no difference in markers of cerebral or myocardial injury, EEG findings, and neurologic outcome [50]. 

## 6. Improving Contractility

Once preload and afterload are optimized, contractility needs to be addressed in order to increase SV further and, therefore, DO_2_. It must be recognized, however, that not all patients need their Frank–Starling curve to be “pushed up.” Each patient has an intrinsically acceptable SV and CO; and there is no universally accepted “normal.” Cattermole et al. used ultrasound to evaluate hemodynamic parameters of over 2000 subjects ranging in age from 1 to 89 years. CO varies among age and weight, and even significantly within age groups [51]. Thus, increasing SV by increasing contractility is not always the answer when trying to optimize DO_2_. Furthermore, before increasing contractility we need to ensure oxygen content is optimized, as increasing contractility increases myocardial oxygen demands. 

## 7. Increasing Arterial Oxygen Content

Arterial oxygen content (CaO_2_) is a product of hemoglobin, arterial oxygen saturation (SaO_2_), and partial pressure of oxygen (PaO_2_). While increasing SaO_2_ naturally increases DO_2_, the ideal oxygen saturation is not known and may vary based on the patient’s co-morbidities and disease process. In patients with traumatic brain injury, a higher PaO_2_ increases oxygen tension, thus optimizing the diffusion of dissolved plasma oxygen into brain tissue [52]. However, studies suggest that we should limit the PaO_2_ levels of critically ill patients within a safe range. A PaO_2_ greater than 200 mm Hg is associated with higher mortality since hyperoxia increases the risk of tissue damage by reactive oxygen species as well as hyperoxia-induced vasoconstriction [53]. In mechanical ventilation, a conservative oxygen therapy approach targeting SpO_2_ 90–97% showed no difference in ventilator-free days [54]. A systematic review and meta-analysis of 25 randomized controlled trials showed that oxygen therapy to SpO_2_ greater than 94% was associated with increased mortality [55]. Thus, a recent clinical practice guideline recommends a target SpO_2_ range 90–94% in most critically ill patients [56]. 

There is still debate surrounding the role of transfusion in shock. The landmark Transfusion Requirements in Critical Care (TRICC) trial randomized 838 critically ill patients to receive either a restrictive (transfuse for Hgb < 7 mg/dL) or liberal (transfuse for Hgb < 9 mg/dL) transfusion strategy [57]. Overall there was no difference in the primary outcome of 30-day mortality, but a benefit with liberal transfusion was shown in those patients under age 55 and patients with APACHE II ≤ 20. Conversely, there was increased mortality in the liberal arm in those patients with ischemic heart disease. The Transfusion Requirements in Septic Shock (TRISS) trial, however, showed similar outcomes related to mortality and ischemic events in liberal versus restrictive transfusion [58]. 

Generally, transfusion will improve oxygen delivery in life threatening anemia, as severe anemia eventually leads to a critical level of oxygen delivery requiring intervention. However, there still remains controversy regarding the degree in which transfusion improves tissue oxygenation in moderate anemia (hemoglobin 7–10 g/dL), since normal physiologic mechanisms such as increased cardiac output and increased oxygen extraction at the tissue level allow for compensation in the setting of moderate anemia. In addition, notable is the importance of maintaining normovolemia when transfusing an anemic patient, as red blood cells increase blood viscosity and have been shown to decrease cardiac output if hematocrit becomes significantly elevated. In such a situation, the additional red blood cells become ineffective transporters of oxygen to the tissue [59].

## 8. Targeting Contractility and Hemoglobin Guided by SvO_2_ or ScvO_2_

**Figure 4:**
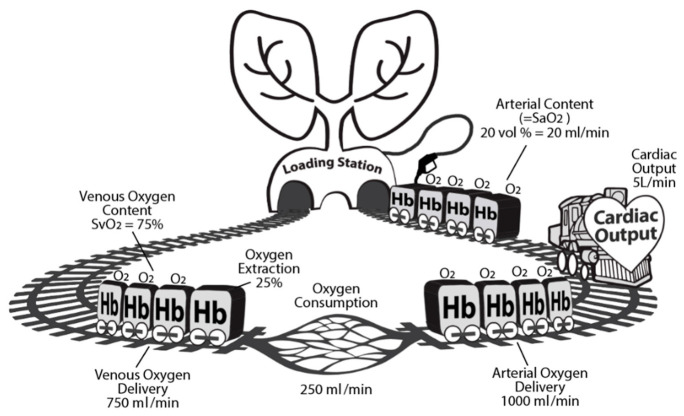
The Oxygen Choo-Choo Train illustrating the relationship between cardiac output, oxygen content, and oxygen consumption.

The decision to initiate inotropic support such as with dobutamine and/or to transfuse can be guided by venous oxygen saturation (SvO_2_) measured at the pulmonary artery. Central venous oxygen saturation (ScvO_2_) measurements, obtained in the superior vena, can be used as a surrogate to SvO_2_ and more feasibly measured with a central venous catheter. ScvO_2_ parallels SvO_2_ but is on average 7 ± 4% higher [60]. SvO_2_ reflects the relationship between oxygen consumption and oxygen delivery, as described by the “Oxygen Choo-Choo Train” model (Figure 4).

The Oxygen Choo-Choo Train model describes the relationship between CO, oxygen content, and the consumption of oxygen (VO_2_) [61]. The engine represents CO, and the train cars represent hemoglobin carrying oxygen for delivery. The “oxygen consumption station” is where oxygen is extracted and consumed at the level of the tissues. After leaving the “oxygen consumption” station, the train then carries venous blood (usually ~75% of arterial oxygen content) back to the lungs or “oxygen loading station”. 

If a patient has low SvO_2_, then there is either a decrease in DO_2_ or an increase in VO_2_. Decreased DO_2_ may be due to anemia, hemorrhage, hypoxia, hypovolemia, or cardiac failure; whereas, increased VO_2_ can be caused by agitation, fever, pain, shivering, respiratory muscle work, or any increase in metabolic demand [62]. In relation to the Choo-Choo Train model, there is either a problem with the train or an increase in VO_2_ at the station. Patients with low SvO_2_ and high CO may be anemic (requiring transfusion) or hypoxemic (requiring mechanical ventilation). In this scenario, the engine (CO) is going at the proper speed, but there are not enough train cars (hemoglobin) to carry oxygen or not enough oxygen on the train cars. At the tissue level, the maximum oxygen extraction ratio is ~50%; thus, if SvO_2_ < 50%, then low DO_2_ must be a contributor, not just high VO_2_. Patients with low SvO_2_ and low CO may be in cardiogenic shock requiring inotropes. In relation to the Choo-Choo Train model, inotropes can be seen as an enhancement to the engine. At the bedside, when SvO_2_ is low, strategies to decrease VO_2_ should be considered first, such as analgesia, sedation, antipyretic, or even intubation, prior to increasing DO_2_ with blood transfusion or inotropes.

## 9. SvO_2_ and Lactate as Indicators of Shock and Resuscitation Endpoints

As discussed, SvO_2_ reflects the relationship between VO_2_ and DO_2_, and helps identify if CO is sufficient to meet metabolic demands. Importantly, SvO_2_ reflects the oxygen deficit that occurs in shock. The concept of the oxygen deficit was first described in 1964 as the integral difference between the rates of oxygen usage before and after the onset of shock [63]. When there is an inadequate DO_2_ to meet oxygen demand (low SvO_2_), or when oxygen utilization is impaired from extraction defects (high SvO_2_), an oxygen deficit develops. It is calculated as a difference between the baseline VO_2_ and VO_2_ measured at a particular time point during shock. Therefore, SvO_2_ represents a snapshot of an oxygen deficit. Continuous monitoring of SvO_2_ allows for minute-to-minute resolution of the ongoing oxygen deficit.

If the oxygen deficit is not addressed and allowed to persist, an oxygen debt develops, or an accumulation of multiple oxygen deficits over time [64]. This occurs when VO_2_ remains persistently low due to inadequate DO_2_, resulting in global tissue hypoxia and lactate production. As a compensatory mechanism, oxygen extraction can increase to maintain VO_2_ while DO_2_ is being optimized. However, a persistently elevated lactate suggests that the oxygen debt is unmet.

**Figure 5:**
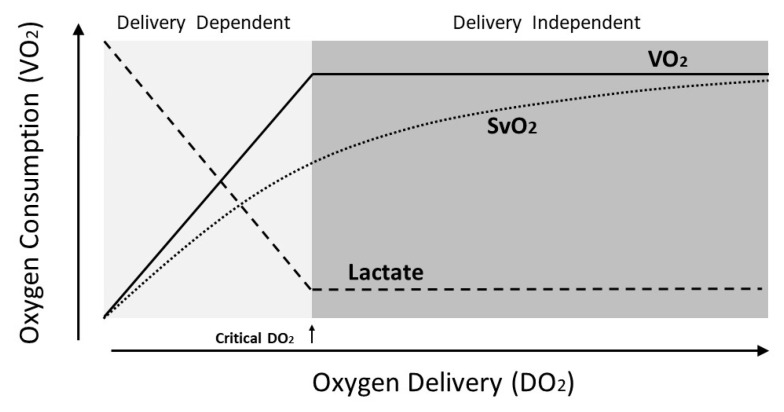
Relationship of oxygen delivery (DO_2_), oxygen consumption (VO_2_), venous oxygen saturation (SvO_2_), and lactate. Below the critical DO_2_ threshold, SvO_2_ decreases and lactate increases, reflecting ongoing oxygen deficit and accumulating oxygen debt.

Based on the above understanding, low SvO_2_ and high lactate levels indicate ongoing shock when DO_2_ falls below the critical oxygen delivery threshold to meet the required VO_2_ in the tissues. Increasing oxygen extraction alone is not adequate, and measures to increase DO_2_ above the critical threshold are necessary (Figure 5).

Monitoring both SvO_2_ and lactate can help the clinician recognize the different states of ongoing shock: (1) A normal SvO_2_ and normal lactate represents a resuscitated patient with adequate DO_2_; (2) an oxygen deficit occurs when SvO2 is low, but lactate remains normal as cells maintain function by increasing oxygen extraction; (3) when increasing oxygen extraction is not adequate, and DO_2_ falls below the critical threshold, lactate increases with persistently low ScvO_2_ signifying an oxygen debt as cells switch to anaerobic metabolism; and (4) if the oxygen debt is not resolved, lethal cell injury occurs with mitochondrial dysfunction, and cells can longer extract and utilize oxygen resulting in high SvO_2_ and high lactate [65].

Decades of literature have shown that elevated lactate in critically ill patients is associated with increased mortality, and the normalization of elevated lactate (lactate clearance) improves outcomes [66]. However, lactate clearance alone is an insufficient endpoint in shock resuscitation, as normal lactate levels may exist in up to 50% of patients with septic shock [67,68]. Therefore, resuscitation should be focused on both the normalization of lactate and SvO_2_. Additionally, a decreased SvO_2_ in the presence of elevated lactate suggests the presence of an oxygen debt rather than other etiologies of lactate elevation without tissue hypoperfusion.

## 10. Liberal versus Restrictive Fluids or Vasopressors

Emerging differences between approaches to septic shock are focused on a *liberal* versus *restrictive* fluid approach. In the restrictive approach, MAP goals are achieved via the early administration of vasopressors. Evidence that supports a liberal fluid approach is based on replenishing intravascular fluid lost to the extravascular space due to endothelial dysfunction seen in sepsis. Replenishing intravascular space thereby increases preload and cardiac output, as discussed in this review. Evidence that supports a more restrictive therapy is based upon the idea that fluid boluses transiently increase intravascular volume, but eventual extravascular fluid shifts occur resulting in pulmonary edema and other complications. Those in support of early vasopressor use aim to decrease venous capacitance and shift unstressed volume to stressed volume, which then drives tissue perfusion. However, vasopressors have potential deleterious effects including organ ischemia and increased myocardial oxygen demand.

Recent studies suggest there is no difference in survival between liberal versus restrictive fluid resuscitation strategies [69,70]. Additionally, the early use of norepinephrine instead of fluids was associated with a lower incidence of cardiogenic pulmonary edema and new onset arrhythmia [69]. However, a restrictive fluid approach may increase the risk of acute kidney injury [70]. The ongoing CLOVERS trial comparing liberal fluids versus early vasopressor in septic shock resuscitation will hopefully provide further evidence and consensus on these strategies [71]. 

## 11. Putting It All Together

In summary, resuscitation of shock includes optimizing preload, afterload, contractility, hemoglobin, and oxygen saturation. This is the fundamental principle behind the Early Goal-Directed Therapy (EGDT) protocol for septic shock [72]. EGDT includes targeting CVP of 8–12 mm Hg (optimizing preload), MAP goals 65–90 mm Hg (optimizing afterload), and ScvO_2_ > 70% (optimizing contractility via inotropes and oxygen saturation/hemoglobin via transfusion or mechanical ventilation), with the goal of restoring tissue oxygenation and repaying oxygen debt within the first 6 h. It is important to realize these concepts are applicable to other types of shock (cardiogenic, hypovolemic) as the goal of therapy is to provide adequate oxygen delivery. This structured approach provides an overall framework for resuscitation with physiologic sound endpoints.

**Figure 6:**
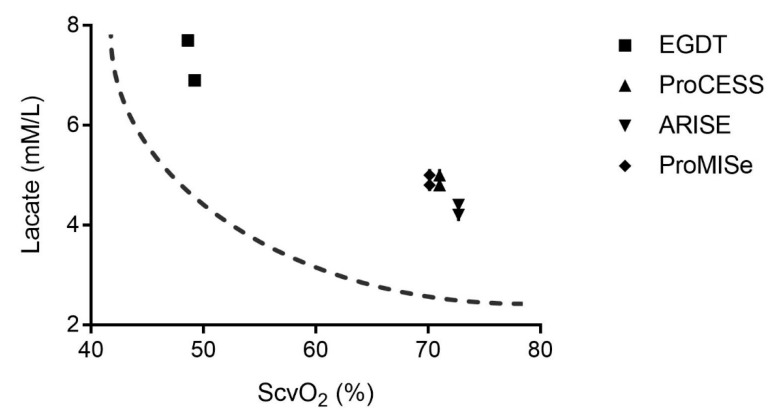
Comparing phenotypes (determined by ScvO_2_ and lactate) of septic shock patients enrolled in studies comparing Early Goal-Directed Therapy (EGDT) versus usual care [76].

Contemporary multi-center clinical trials (ProCESS, ARISE, and ProMISe) have compared standard therapy to EGDT and showed no difference in clinical outcomes in septic shock [73,74,75]. Unfortunately, these results have led to a sea change of clinicians abandoning the fundamental principles of oxygen transport as it relates to shock management and initial resuscitation. For septic shock, treatment at the bedside has been minimized to “usual care”. We are not clear what usual care means or how to teach it to the next generation of clinicians. However, on further examination, these aforementioned trials have also taught us that different hemodynamic phenotypes exist in septic shock, reflected by the ScvO_2_ and lactate levels of patients enrolled (Figure 6). 

Perhaps, shock resuscitation with the goal of optimizing DO_2_ is beneficial in only patients who have low ScvO_2_ (or SvO_2_) and high lactate with significant oxygen debt, such as those patients enrolled in the original EGDT trial. On the contrary, patients enrolled in ProCESS, ARISE, and ProMISe (a.k.a. the Trio Trials) had normal ScvO_2_, albeit elevated lactate. While a meta-analysis of the Trio Trials did not show benefit with EGDT in various subgroups of illness severity, there was no comparison between EGDT versus usual care for the subgroups of low, normal, and high ScvO_2_ [77]. Given our understanding of shock, this different phenotype in the Trio Trials may not have an oxygen deficit nor oxygen debt. Applying the physiologic principles discussed here, one can critically examine the evidence presented in these and future shock resuscitation trials. Were patients truly in shock or already partially resuscitated? In those patients without ongoing oxygen deficit, usual care without targeting all the components of DO_2_ may be adequate. Thus, it was not surprising that EGDT did not show any outcome benefit in the Trio Trials. However, the phenotype with significant oxygen debt when examined by Protti et al. has shown outcome benefits almost identical to the EGDT trial [78]. In the ALBIOS trial, all patients received EGDT to target ScvO_2_ > 70%. Persistence of low ScvO_2_ was associated with higher 90-day mortality, possibly because it reflected underlying cardiac dysfunction. The authors concluded that subjects with ScvO_2_ < 70% may benefit most from interventions aimed at normalizing the balance between systemic oxygen delivery and consumption.

While EGDT may not be effective in the less sick patients, we should not forget the concepts of shock resuscitation presented in this review. Focusing solely on preload and its responsiveness without addressing the other components of DO_2_ in a severe shock patient is intuitively inappropriate. Shock will only progress to increasing lactic acidosis, multi-organ failure requiring multiple vasopressors, and death. Shock treatment should not be minimized to a mere passive leg raise and a debate on when to give the fluid bolus, but rather be considered in the broader context of optimizing oxygen delivery to meet the oxygen demand with appropriate assessment of the different hemodynamic components.

We will conclude our review with a case example to illustrate how we can apply the various technologies available to treat a patient with septic shock optimally.

### Case Example

A 67-year-old female with a past medical history of diabetes, hypertension, end-stage renal disease on dialysis, and congestive heart disease presented to the emergency department complaining of fever and productive cough for the last week. Her primary physician prescribed her a “Z-pack” 5 days ago, however, her symptoms did not improve. Her vital signs included temperature 38.3 °C, heart rate 132 beats per min, respiratory rate 28 breaths per min, blood pressure 84/43 mm Hg, SpO_2_ 92%, and weight 85 kg. Her physical exam included significant bibasilar crackles, otherwise unremarkable. Her laboratories showed a white blood cell count 18,300 per µL, hemoglobin 7.9 g/dL, platelets 156,000 per µL, creatinine 4.6 mg/dL, and glucose 232 mg/dL. Lactate was 7.4 mmol/L and procalcitonin 4.3 ng/mL. Her chest radiograph showed bilateral lower lobe consolidations consistent with severe pneumonia.

Given the patient’s significant tachycardia and hypotension, an immediate 30 mL/kg crystalloid fluid bolus was given via a peripheral intravenous catheter. Her blood pressure improved to 90/52 mm Hg (or MAP 65 mm Hg). Without vasopressor administration requiring a central venous catheter yet, monitoring preload and assessing fluid responsiveness can be achieved with ultrasound measurements of the IVC collapsibility. Alternatively, the bioreactance technology can be used to monitor SV and CO. A PLR maneuver or an additional crystalloid fluid bolus of 500 mL can be administered to determine fluid responsiveness by calculating the change in stroke volume index (∆SVI) before and after the PLR or fluid bolus.

After 2 additional liters of crystalloid fluid boluses, the patient became more hypotensive with blood pressure 78/45 mm Hg (or MAP 56 mm Hg). A central venous catheter (CVC) with continuous ScvO_2_ capability was inserted in her right subclavian vein. The subclavian vein approach was chosen over the internal jugular vein since the catheter can be inserted quickly without the need of ultrasound guidance. An arterial catheter was inserted in her left radial artery. Norepinephrine was initiated and the dose was quickly increased to 15 mcg/min. With the CVC in place, CVP monitoring should be started. Her CVP was 6 mm Hg with ScvO_2_ 55%. Her recent ∆SVI was 18%. Additional 2 L of fluids were administered to reach CVP 10 mm Hg, MAP 68 mm Hg, and ScvO_2_ 58%. Her heart rate also decreased to 95 beats per minute. 

Her CO on the bioreactance monitor was 3.8 L/min, with ∆SVI 8% after the most recent fluid bolus. With the arterial catheter in place, an alternative technology of arterial waveform analysis could be used to monitor CO; however, we decided to continue with bioreactance CO measurements. If the patient required intubation and mechanical ventilation, then SVV monitoring with arterial waveform analysis to determine fluid responsiveness may be preferred over repeated measurements of ∆SVI determined by PLR maneuvers.

At this point, the patient’s hemodynamic status seemingly appeared stable on norepinephrine and was no longer fluid responsive. However, her ScvO_2_ remained low. A repeat lactate measurement was 5.6 mmol/L. Given ScvO_2_ < 70% and lactate > 4 mmol/L, we targeted a higher hemoglobin > 9–10 g/dL with transfusion of 2 units of packed red blood cells. After transfusion her ScvO_2_ increased to 63%. The persistently low ScvO_2_ and low CO required us to initiate dobutamine at 5 and then 10 mcg/kg/min. Her ScvO_2_ then normalized to 72%, with CO 6.2 L/min. The patient then met her resuscitation endpoints with another repeat lactate 2.8 mmol/L, showing a lactate clearance of 62%. She was admitted to the intensive care unit. Cultures showed that the patient had Klebsiella pneumonia. Norepinephrine and dobutamine were titrated off within 48 h, and patient was discharged home after 5 days.

In summary, based on our application of oxygen transport physiology at the bedside, we utilized the available hemodynamic monitoring tools to optimize CVP, MAP, CO, ∆SVI, and ScvO_2_ in order to increase DO_2_ and resolve the oxygen debt present in septic shock. Without this comprehensive approach, the patient would not have received the required amount of fluids, vasopressor, blood transfusion, and inotrope. Performing repeated PLR maneuvers may address the inadequate preload with a transient decrease in lactate. However, if a vasopressor is initiated without resolving the imbalance between DO_2_ and VO_2_ reflected by a decreased ScvO_2_, oxygen debt and lactate elevation will continue. Shock will only progress to multi-organ failure and death.
